# Pyorrhea Alveolaris from a New Standpoint

**Published:** 1897-03

**Authors:** William H. Trueman

**Affiliations:** Philadelphia.


ARTICLE III.

### PYORRHEA ALVEOLARIS FROM A NEW STANDPOINT.

BY WILLIAM H. TRUEMAN, D. D. S., PHILADELPHIA.

In a paper read at the last meeting of the Kentucky State Dental Association,[1] Dr. Junius E. Cravins, who for a long time has made a close study of pyorrhea alveolaris, advanced somewhat novel ideas bearing upon this disorder. Accepting the theories advanced by two recent medical writers[2] upon conditions which he assumes are closely allied to pyorrhea alveolaris, he has modified somewhat the treatment he suggested in his treatise upon this subject,[3] published two years ago, and now recommends a procedure very closely resembling that advocated by Thomas Berdmore, a distinguished dentist of London, in a work he published in 1760;[4] so closely, indeed, that we are tempted to ask, Have we, in the intervening century and a quarter, simply swung around a circle?

The treatment recommended by Dr. Cravens in his recent paper, briefly stated, is this: After removing all deposits from the affected roots, he lacerates or even tears away the granulation tissue within the pockets. Then applies an astringent stimulant of acidulated nature. This is followed, after sufficient time has elapsed for granulations of repair to be well set up, by an astringent stimulant of a more pronounced and permanent effect. The object of this treatment is to establish scar tissue. He assumes

1 "Pyorrhea Alveolaris; or Riggs's Disease; Etiology, Pathology, Characteristic Treatment." Read at the twenty-sixth annual meeting of the Kentucky State Dental Association at Louisville, June 19–18, 1896. The *Dental Digest*, Vol. II., August, 1896, p. 435.

2 Dr. R. H. M. Dawbarn, in the "Reference Hand-Book of the Medical Sciences"; Greene's "Pathology and Morbid Anatomy"

3 "A Treatise on Pyorrhea Alveolaris." James E. Cravens, D. D. S., Indianapolis, 1894.

4 "Treatise on the Disorders and Deformities of the Teeth and Gums." By Thomas Berdmore. London, 1768: Dublin, 1769; second edition, London, 1770.

that pyorrhea alveolaris is a periostitis, and advances the name, alveolar periostitis, as proper and fitting for this affection. The cure of periostitis, he says, demands the establishment of scar tissue, basing this assumption, he explains, upon certain statements by Dr. R. H. M. Dawbarn, in a chapter upon periostitis, ostitis, etc., in the "Reference Hand-Book of Medical Science," and also certain statements in Greene's "Pathology and Morbid Anatomy," both recent publications.

Berdmore says,[5] treating of a condition which we readily recognize as pyorrhea alveolaris of to-day. "The treatment is partly medical and partly surgical. The former consists in removing the original disease of the whole body by a due course of medicine, and in washing the mouth frequently with antiseptic and astringent liquors, rendered slightly acid by means of orange, lemon or sorrel juice or vinegar. The surgical treatment consists in scarifying and pricking the affected gums, and destroying their tender outer skin in such a manner as to occasion a fresh shooting forth and elongation of their substance, and such a solidity as will endure the usual impressions of mastication. When the gums have lost their connection with the teeth, or when they do not embrace them closely, cutting a small slip away from the forepart is of considerable service, for the new gum will then adhere to the tooth, or at least will embrace it more closely." During the time necessary for completing the cure in this manner opiates, solution of camphor, or a few drops of nitrous ether in common spirits may be used to mitigate or remove the pain. He explains that sometimes one will give relief, and at other times the other. He recognizes the importance of first thoroughly cleaning the teeth of all deposits, and states that the scarifying or cutting of the gum may have to be frequently repeated. He relates a case,[1] where "the incisors of both jaws were entirely naked to the extremity of each

5 *Ibid.*, edition 1770, p. 66.

1 Berdmore, edition 1770, p. 70

root," that required five or six operations, stating that after a perseverance of six weeks the gums were completely restored, and have remained sound ever since by the assistance of astringent washes and brushing. Berdmore claims that when the patient will submit to the necessary treatment and follow closely his directions, the surgeon-dentist will seldom fail of success in cases of this kind. Teeth that are very loose, or badly injured by caries, he recommends should be extracted, that being for such teeth the only cure.

This close identity of the latest suggested treatment of pyorrhea and that in vogue so long ago is quite suggestive, and well deserves your careful attention. I say, in vogue so long ago, for Berdmore does not claim this treatment as his; indeed, it seems to have been the common practice long before his advent into the dental world.

Unanimity of experience in treating this disorder seems to have been in that day, as in this, sadly wanting. Robert Wooffendale, writing in 1783, referring to the treatment recommended by Berdmore, and to the case Berdmore especially reports, says,[2] "Such cases I have frequently seen, but never cured one, or saw one of the same kind, or anything like it, cured by any other person." He further says, "Lancing the gums, to prevent the scurvy in them, is with some people a fashionable operation, and which they have performed regularly once a month; some once a week, or oftener; supposing it will prevent or remove all complaints of the gums, teeth, and their connections. By obobservation, however, this operation performed in such manner by no means proves such expectations well founded. The operation, frequently repeated, may be lucrative to the operator; but in my humble opinion is of little, if any advantage to the teeth or gums of the patient."* He recommends to prevent this condition the daily use in the

---

2 "Practical Observations on the Human Teeth." By Robert Wooffendale, London, 1783; p. 114.

* "Practical Observations on the Human Teeth." By Robert Wooffendale, London, 1783; p. 111.

morning of an astringent lotion, taking care to have any small portions of tartar that may adhere to the teeth under the edges of the gums removed by the instrument of a careful dentist, and the frequent use of a proper dentifrice so as to keep them as clean as possible. Where this treatment is strictly attended to he believes the disease will very seldom make its appearance, and in recent cases it is generally sufficient to prevent its farther progress.

When through long neglect the disease has made progress, but not yet reached the incurable stage, it is generally proper, he says, "to lance the gums, and sometimes to repeat it daily for a fortnight, a month, or longer. When the gums are much thickened by this disorder considerable portions of them should be cut off, which in many cases I have done, and the patient has not been sensible of the least pain till the operation has been repeated several times."

Robert Wooffendale was a student of Thomas Berdmore. I note here and there throughout his book a disposition to give his preceptor a sly rap now and again. I note also, in dental literature of recent date, this disposition is still manifested by some of our profession who fail to see matters in the same way their brethren do. I still further note that this disposition at times is a serious clog to real progress, in that it provokes antagonism, fosters an unreceptive spirit, and prevents that bringing together, careful sifting, and equitable comparison of our varied experiences, by which process alone can they be made practically useful.

Berdmore considered this disorder curable, even when it had far progressed, and claimed as part of the cure a reproduction of lost tissue. Wooffendale that it is usually curable, so long as there remained embedding the roots of the teeth enough tooth-supporting tissue to securely hold them. He contends that to restore to health the disordered tissues is all that can be expected; and both agree that to retain them in this condition requires constant care and continued watchfulness on the part of the patient. Wooffendale further says, and this with emphasis,* "When the exposure of the root is occasioned by accident, as a bruise, a cut, or the like, it will frequently be readily restored by Nature, generally without the assistance of art, but when the smallest part of the roots of the teeth are exposed, in consequence of the adhesion of tartar on them, by the scurvy in the gums, veneral infection, or the imprudent use of mercury, I never saw the least disposition of the gums to grow to the teeth, although assisted by scarification, or by stimulating, balsamic, astringent, or any other sort of washes or applications; the gums would as soon grow to a piece of ivory or iron as to the root of a tooth which has lost its periosteum from any of the causes here alluded to." Jourdian writes dolefully of this disorder and says,† "Those who think they have made cures by scarifications, etc., by cautery, issues, and the like, have confounded this with suppurative fungus of the gums alone. I have seen many cases treated by these so-styled successful remedies, and I may safely say I have yet to see the first cure performed."

How very like the remarks in reported discussions on this subject, in this year of grace eighteen hundred and ninety-six, are these, culled from writers of more than a century ago, writers who were well conversant with, and who have accurately described, this and other allied disorders of the teeth and gums, that in their day, as in ours; in spite of their efforts and in spite of our efforts, result in a much-to-be-deplored tooth loss. With them, as with us, whether these disorders were merely local, or were wholly or in part systemic, was a much debated question; indeed, a careful study of the accurate and full records they have left makes one ponder and prompts the question, What do we know of these disorders that they did not know?

---

* "Practical Observations on the Human Teeth," p. 107.

† "Diseases and Surgical Operations of the Mouth and Parts Adjacent." M. Jourdian, Paris, 1778. Translation published by the American Society of Dental Surgeons, Baltimore, 1849, p. 352.

When, where, or by whom the term pyorrhea alveolaris was first used I do not know. The older writers described the conditions we usually associate with that expression under various names,—scurvy of the gums, recess of the gums, erosion of the gums, conjoined suppuration of the alveoli and the gums, etc. At that day, as in this, writers were not careful to use exact terms in describing nearly allied pathological conditions, and no doubt then, as now, this was a factor largely controlling success or failure of treatment.

Most earnestly would I urge upon those making a special study of this and allied disorders, a thorough research and careful study of its literature. I question if any department of our art has been more carefully studied or more fully written up than has been the disorders and diseases of the tissues surrounding the teeth. Faucnard, Bourdet, Ricci, and other able writers and observers made of the diseases of the gums and the effects of systemic disorders upon the gums and teeth a careful study. They visited the hospitals and in conference with physicians and surgeons earnestly and zealously labored to master the same conditions that confront and baffle us of to-day. Many writers of to-day, giving the results of their recent studies in this field, would probably be astonished were they to see their productions in parallel column with those of Bourdet; Jourdain, and many others who lived and labored before the present century began. When a writer expresses the opinion that this disorder is becoming increasingly frequent; that it is the outcome of modern ways of living; that it is largely more prevalent as the result of imperfectly performed dental operations, I wonder if he knows that more than a century ago it was so prevalent, so intractable to treatment, so prolific of tooth loss, that the dentists of that day, discouraged and disheartened, labelled it devastation of the teeth. Of it D. H. H. Hayden, writing in 1822, has this to say :*

*_American Journal of Dental Science_, Vol. II., March 1842, page 229.

"This disease, from the nature and extent of its ravages, as great or more so among the opulent and rich as among the poorer classes of society, has, at different times, engaged the attention of some of the most skilful physicians, as well as professional dentists, in Europe; and, in the course of treatment which they have pursued, they have, severally, resorted to every means for its cure that medical skill could suggest,—such as emollient, astringent, and detergent gargles of various kinds; astringent, tonic, and antiseptic elixirs; mercurial washes, absorbent powders, aromatic pastes, electuaries, alteratives, sedatives, venesections, vesicatories, injections, setons, issues, excision of the diseased parts scraping the diseased bones, repeated applications of the actual and potential cautery, etc.,—notwithstanding which, their efforts have proved ineffectual."

Scurvy, scrofula, catarrh, syphilis, malnutrition, rheumatism, gout, mental diseases, care, worry, anxiety, etc., are among the systemic conditions or disorders that have been credited with its causation, while now and again various remedial agents have been held responsible for the mischief it has wrought. Some of the older writers have observed that persons subject to gout or rheumatism were seldom afflicted with this disease; on the contrary, those afflicted with suppuration of the gums are seldom troubled with gout or rheumatism; while recent writers contend that gout, rheumatism, and phorrhea have a common origin and are frequently associated. From the earliest mention of this disorder to the present hour, regarding it, our profession has been quite unsettled, and, as a natural result, much time and labor has been expended in rehabilitating discarded theories or in the effort to supplant one unsatisfactory theory by another equally at variance with observed facts. Fauchard was inclined to reject the theory that certain diseased conditions of the system were the cause of it, because he had frequently seen cases where the systemic diseases were well marked and this trouble did not exist; and also from having observed the converse of this, cases where the trouble was well marked and no systemic disease was present; and the further observation, that remedies suited to those diseases had no effect whatever upon the oral trouble. He regarded the coexistence of the two as a mere accident, and was more firmly inclined to this opinion from having frequently observed that in such cases, that while the systemic trouble readily yielded to appropriate remedies, there was no corresponding improvement in the oral lesion. Systemic treatment, he was impressed, was of little avail. Precisely the same statements have been made at a very recent date, while not universally accepted. I am impressed that Fauchard's position in this matter is substantially that of the masses of the profession at this time.

This brings us to the important question, concerning pyorrhea alveolaris, What do we really know? Upon what points do those who have carefully studied it universally agree? There are, it is a satisfaction to know, a few points upon which there has been little if any controversy; and these may, perhaps, furnish us reliable data for future investigations.

1. It is a disease seldom appearing until the patient approaches middle life. While exceptions to this have been frequently noted, they are, however, infrequent and exceptional, and have been so recognized.

2. It is not, at the onset, usually accompanied by any marked systemic derangement.

3. It usually begins insiduously, runs an uneven course, and is prone to relapse.

4. It always ceases, completely and permanently, when the teeth are lost.

I note that now and again observers have remarked the close resemblance physically, and the very marked differences pathologically, between pyorrhea and that acute inflammation of the gums due entirely to systemic conditions, known as stomatitis. This is ushered in by well-marked and unmistakable systemic derangement, quickly reaches its height, responds promptly to systemic treatment, and, as a rule, in mild cases leaves the involved oral tissues but little the worse for its presence. When at its height, this condition resembles closely, so far as it affects the oral cavity, pyorrhea at its worst. We have the same turgid and swollen gum margins, the same profuse flow of pus from the alveoli, the same fetid odor, and the same loosening of the teeth. Stomatitis, however; is a disease of childhood; pyorrhea, of mature age. Stomatitis comes on quickly, the acute and violent oral symptoms are merely the expression of a more profound systemic derangement, —a systemic derangement so profound, indeed, as to frequently compromise life. In a mild, uncomplicated attack, it quickly runs its course, and the oral symptoms disappear as quickly as they came. It is very probable that the oral symptoms linking together stomatitis and pyorrhea are alike due to germ-infection, the sudden and violent onset in stomatitis being due to quickly-developed conditions favoring germ activity, and the rapid subsidence in mild cases, it has been explained, is due to the violent inflammation proving fatal to germ-life. In severe cases so violent is the inflammation that gangrene, with all its attendant seriousness, not infrequently supervenes. In adults the gums are not so responsive to systemic disturbances, nor yet do slight disorders of the nutritive functions, as in early childhood, so quickly or so profoundly affect the general health. Under these conditions stomatitis becomes far less frequent and far less serious. It still, however, preserves its well-marked characteristics distinguishing it from pyorrhea alveolaris, and these may have value in determining the cause and character of the latter disorder. I am strongly impressed that, after all, pyorrhea alveolaris is merely a germ-infection. It may be serious, involving a large territory, and incurable, or slight and easily controlled, just in proportion as the oral conditions favor or repress germ activity. How far the general health may affect these conditions we have, as yet, but little reliable data. The pathology of stomatitis is suggestive upon this point. We may bear in mind that for the germ to become a pathological factor three things are necessary: (1) the germ; this, however, is omnipresent; (2) a dwelling-place; (3) congenial surroundings. In stomatitis the disturbed circulation, due to the primal lesion resulting in the swollen gum margins, furnishes over a widely extended territory abundant opportunity for germ colonies, and the surroundings seem particularly favorable to germ activity. Disordered digestion in the child and in the adult seems to produce in the oral cavity conditions especially favorable to the germ-growth. In proof of this, the furred tongue, for ages recognized as a reliable and delicate indication of gastric disturbance, is now known to be due to the presence of germ-life,—a form of germ-life that immediately disappears when the stomach resumes its normal condition.

In early life, while the gum-tissues firmly embrace the teeth and fully occupy the dental interspaces, there is little opportunity for any form of germ-life to obtain a permanent foothold. Later, however, when normal changes materially alter this condition of affairs, when the saliva becomes more loaded with inorganic matter, when there is a more marked disposition to tartar deposits, when the gum-tissues relax their firm embrace of the teeth and as a result debris-collecting spaces are formed along the gum-marging and between the teeth, when, in addition to all this, as the natural result of normal wear and tear and the inevitable loss of tooth-substance, the gum- and tooth-supporting tissue becomes more and more exposed to violence and accidents of various kinds during mastication, etc., we readily see how, in a thousand and one ways, opportunities for germ infection are vastly increased. All without the slightest assistance from any systemic disorders.

We may remember, also, that of all germicides known to science none are so potent, so thoroughly reliable, or so universally accessible as are the natural secretions of a healthy human mouth. Were it otherwise, it would be utterly impossible to find a healthy individual among the many thousands who are compelled to daily breathe the germ-laden dust of a large city. It is only when the germ succeeds in quickly finding a little pouch in which to hide, secure from this, to it, destructive secretion, or when by mere chance it obtains foothold while the potency of this secretion is temporarily impaired, that it has the slightest chance to begin its destructive work. Once firmly settled, it begins to thrive, and a colony once formed creates conditions that may successfully combat its untoward surroundings. Changes constantly taking place in the oral secretions—now favoring and now opposing—fully account for the ebb and flow that marks, in its earlier stages, this disorder. The ravages of the disease, never wholly obliterated after it has once been firmly established, are ever an open door inviting the frequently noted relapses, until the complete loss of the teeth destroys forever its once favorite camping ground.

We thus see how fully the germ theory meets all of the few points upon which close observers for nearly two centuries are in perfect accord.

I present it to your notice, not as a new theory, but as a theory fully in line with modern thought, and so fully in accord with observed facts as to warrant its selection as a new standpoint from which to study the cause and treatment of pyorrhea alveolaris,—a standpoint much to be preferred to those older theories with which, as I have shown you, our grandfathers labored so earnestly and so long, with so little profit.—*International Dental Journal.*

